# Gene expression analysis of Schizophrenia

**DOI:** 10.6026/9732063002001441

**Published:** 2024-11-05

**Authors:** Gunjan Sharma, Ansh Malik, Satyendra Tripathi, Vishwajit Deshmukh, Ashlesh Patil

**Affiliations:** 1 AIIMS Nagpur, India; 2 Department of Biochemistry AIIMS Nagpur, India; 3 Department of Anatomy AIIMS Nagpur, India; 4 Department of Physiology AIIMS Nagpur, India; 5 Bioinformatics Data Analysis Unit (BDAU), AIIMS, Nagpur, India

**Keywords:** Schizophrenia, Prefrontal cortex, Gene expression, Biomarkers, Dysfunctional immunity

## Abstract

Schizophrenia is a chronic psychiatric disorder marked by cognitive deficits associated with prefrontal cortical dysfunction, particularly in Broadmann Area 10 (BA 10), where gray matter reduction is observed. The genetic mechanisms behind these abnormalities remain unclear. Therefore, it is of interest to analyze altered gene expression and pathways in the prefrontal cortex of schizophrenia patients. We used two GEO datasets - GSE12654 (discovery) and GSE17612 (validation) and differential gene expression was assessed between schizophrenia patients and healthy controls. Validation confirmed three upregulated genes (S100A9, S100A8, BCL2A1) and one downregulated gene (CBLB), with protein interaction analysis revealing that upregulated genes were linked to immune and apoptotic processes, while downregulated genes suppressed EGF pathways. These findings suggest immune dysfunction and gray matter loss in schizophrenia, highlighting potential biomarkers and therapeutic targets.

## Background:

Schizophrenia (SCZ) is a chronic psychiatric disorder with a multifaceted etiology, involving genetic and neurobiological factors that affect brain development during early life stages [1, 2]. It presents with a range of symptoms, including hallucinations, delusions and disorganized thinking, along with impairments in motivation and cognitive function [3]. Over the three decades from 1990 to 2019, the raw prevalence, incidence and burden of schizophrenia have significantly increased globally, with a 65% rise in prevalence, a 37% increase in incidence and a 65% surge in Disability-Adjusted Life Years [4]. Despite the potential for patients to improve functioning with current pharmacological and non-pharmacological treatments [2], the lack of a definitive cure for SCZ underscores the need for research aimed at reducing the burden of the disease. Understanding the pathophysiology of SCZ is crucial to achieving this goal. The involvement of prefrontal cortical circuitry dysfunction has been identified as a significant factor in the manifestation of schizophrenia [5]. Dorsolateral prefrontal cortex (DLPFC) that plays a key role in cognitive functioning of the brain including executive functions, working memory and decision making and emotion regulation is often implicated in SCZ [5]. Findings from multiple studies have implicated dysfunction of the DLPFC as playing a central role in the pathophysiology of SCZ [6]. It has been shown in studies that patients with SCZ had significantly reduced overall grey matter volume in prefrontal cortex compared with the controls [7, 8-9]. Research has highlighted excessive reductions in grey matter density in the anterior region of the prefrontal cortex, particularly in the left superior frontal area, including Brodmann area 10 (BA10), among patients with SCZ [10]. During adolescence, a critical period before the typical onset of both bipolar disorder and schizophrenia, this region experiences substantial grey matter pruning [11]. Dysfunction in BA10 is thought to play a role in the common symptoms seen in these conditions. Therefore, to understand the genetic underpinnings of prefrontal cortex dysfunction in SCZ, we explore the up-regulation and down-regulation of RNAs and proteins in the prefrontal cortex and their roles in disease pathophysiology.

## Methodology:

## Data selection and characteristics of discovery and validation datasets:

This study utilized two independent datasets from the Gene Expression Omnibus (GEO) database to investigate gene expression profiles associated with schizophrenia. The selected datasets, GSE12654 [12] and GSE17612 [13], were chosen for their focus on gene expression in Brodmann Area 10 (BA10) of the prefrontal cortex, a brain region implicated in the cognitive deficits observed in schizophrenia. In both datasets, the schizophrenia cases and controls were between the ages of 25-70 years, representing a middle-aged population. The controls were age-matched to the schizophrenia patients, ensuring comparability and reducing potential con founding effects related to age. The study utilized two distinct datasets for analysis, focusing on post-mortem prefrontal cortex samples from Brodmann Area 10 (BA10). The first dataset, GSE12654, served as the discovery dataset and included samples from 13 schizophrenia patients (age 44 ± 14 years, 5 females) and 15 age-matched healthy controls (age 48 ± 11 years, 6 females). These samples were provided by the Stanley Foundation Brain Collection and the diagnoses of schizophrenia were made according to the criteria outlined in the Diagnostic and Statistical Manual of Mental Disorders (DSM). For validation, the GSE17612 dataset was used, which included 7 schizophrenia patients (age 49.5 ± 14.35 years, 2 females) and 9 age-matched healthy controls (age 48.8 ± 14.91 years, 2 females). The samples in this dataset were collected through a prospective collection program coordinated by Imperial College London. Only samples within the age range of 30-60 years, consistent with the discovery dataset, were included in the analysis. Like the discovery dataset, all patients in the validation dataset were diagnosed according to DSM criteria and age-matched controls were selected accordingly. In both datasets, only data from BA10 were analyzed.

## Microarray procedure:

The microarray procedure for both the discovery and validation datasets followed similar protocols, utilizing BA10 brain tissue samples to investigate gene expression profiles. Total RNA was extracted from 0.1 g of frozen BA10 tissues using Trizol reagent (Invitrogen, Groningen, The Netherlands) for the discovery dataset and similarly, using a Polytron-type homogenizer (Yellow Line DI 25 Basic) and Trizol reagent (Invitrogen, Paisley, UK) for the validation dataset. The RNA was further purified using RNeasy columns (Qiagen, Hilden, Germany; Qiagen, Valencia, CA), including an on-column DNase-1 treatment to eliminate any contaminating DNA. For the discovery dataset, the purity of the extracted RNA was assessed using optical density (OD) measurements, while its integrity was confirmed through denaturing agarose gel electrophoresis. In the validation dataset, RNA quality was primarily evaluated using the RNA Integrity Number (RIN), determined by an Agilent 2100 Bioanalyzer (South Plainfield, NJ, USA). Samples were classified into three quality groups-pass (RIN > 7.0), borderline (RIN 6.0-7.0) and fail (RIN < 6.0)-with only "pass" and "borderline" samples being included in the subsequent analyses. In both datasets, microarray analysis was conducted using Affymetrix platforms. For the discovery dataset, 8 to 10 micrograms of total RNA were used to synthesize complementary DNA (cDNA), which was then used to generate biotinylated complementary RNA (cRNA). This cRNA was fragmented and initially applied to the Test 2 Chip (Affymetrix) to evaluate sample quality. Subsequently, it was applied to the HU95A chip (Affymetrix), which contains probes for approximately 12,000 genes. In the validation dataset, 10 µg of total RNA from each batch was processed to produce biotin-labelled cRNA, which was hybridized to HG-U133_Plus_2.0 GeneChips® following the manufacturer's protocol. For both datasets, the hybridization signals were scanned using Affymetrix GeneChip Scanners (HP GeneArray scanner for discovery; GeneChip Scanner 3000 for validation). The scanned data were then processed and analyzed using GeneSuite software (Affymetrix). The consistency in protocols across both datasets ensured that any differences in gene expression could be attributed to biological rather than technical variability, thereby strengthening the validity of the findings.

## Data analysis:

The Data analysis was done using GEO2R, an interactive web tool provided by the GEO database for differential expression analysis. GEO2R utilizes several R packages from the Bioconductor project to facilitate the analysis of high-throughput genomic data. The discovery dataset was initially processed by Experimenter 1. Gene expression profiles were compared between schizophrenia patients and age-matched healthy controls using an unpaired t-test. The analysis of the validation dataset was performed in a blinded approach by Experimenter 2. U133_Plus2 Affymetrix chips were used for mRNA expression analysis and the "affy" package in the R statistical software was employed for data analysis. The analysis comprised three main steps: (i) background correction, (ii) normalization using Robust Multi-array Average (RMA), pairwise comparison and Benjamini-Hochberg False Discovery Rate (FDR) correction and (iii) expression calculation. Following the computation of mRNA expression intensity, a two-tailed t-test with a significance level of p ≥ 0.01 was applied to filter out significantly expressed genes.

## Protein interactome network analysis and functional categorization:

To analyze the derived gene set, the construction of the protein interactome network was carried out using the STRING v12.0 database (https://string-db.org/) [14]. This tool enabled the visualization and exploration of protein-protein interactions within the gene set. A full STRING network analysis was performed, where the edges represented both functional and physical protein associations. The line colours indicated different types of interaction evidence and a high confidence interaction score of 0.9 was set to ensure reliable associations. The network was configured with a maximum of 10 interactors in the first shell and 5 interactors in the second shell to focus on the most relevant connections. After constructing the network, k-means clustering was applied to group proteins into defined clusters based on their centroids, facilitating the identification of key interaction hubs within the network. Following the network analysis, the significantly altered genes were categorized based on their molecular functions and biological pathways. For this, the PANTHER 19.0 (Protein Analysis through Evolutionary Relationships) Classification System (http://www.pantherdb.org/) was used [15, 16]. These bioinformatics tools helped classify the genes and provided insights into their roles in various molecular functions and pathways, thereby deepening the understanding of their biological significance.

## Results:

## Identification of consistently dys-regulated genes in schizophrenia across discovery and validation datasets:

**Figure 1:**
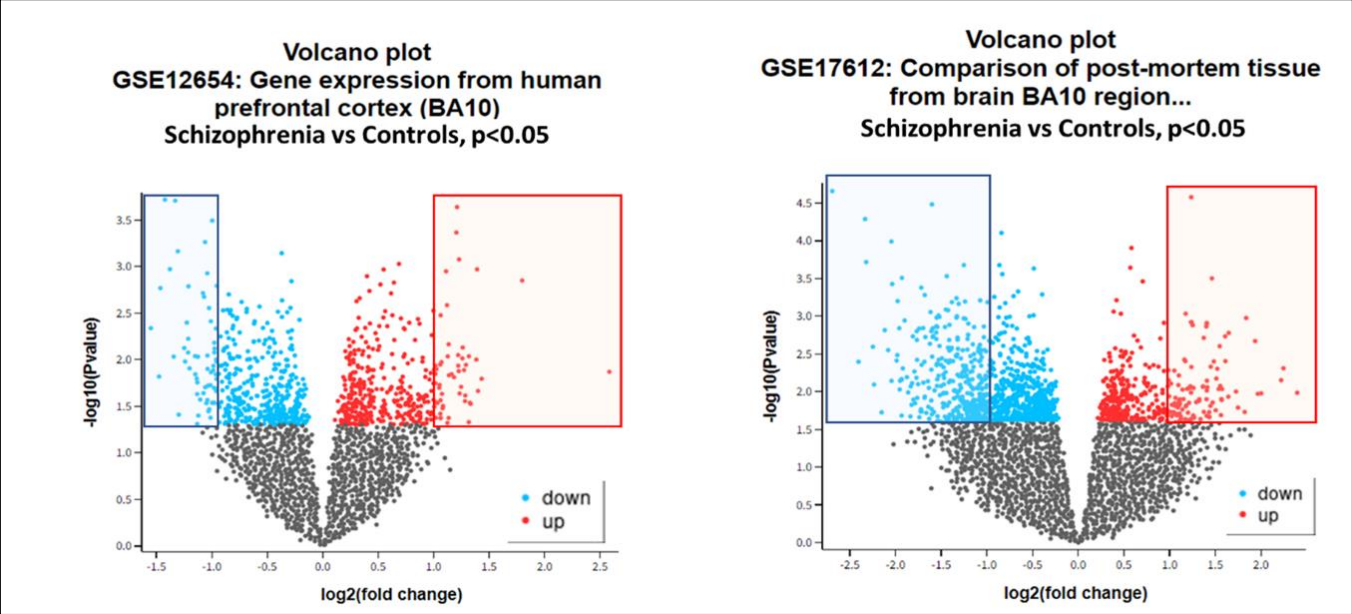
The volcano plot was created using LogFC (fold-change) values and p-values. The blue and red bordered boxes indicate a fold-change of more than 1.0 in both upregulation and downregulation directions, while the horizontal green line represents p-value of 0.05, indicating statistical significance.

In the discovery dataset (GSE12654), 83 out of 12,625 genes were found to be significantly upregulated or downregulated. Among these, 45 genes exhibited more than a 1-log fold change in upregulation, while 38 genes showed more than a 1-log fold change in downregulation (Figure 1). In the validation dataset (GSE17612), 495 out of 54,675 genes were significantly upregulated or downregulated. Specifically, 124 genes displayed more than a 1-log fold change in upregulation and 371 genes exhibited more than a 1-log fold change in downregulation (Figure 1). When comparing the discovery and validation datasets, three genes-S100A9, S100A8 and BCL2A1-were consistently found to be significantly upregulated in schizophrenia patients across both datasets. Additionally, one gene, CBLB, was significantly downregulated in schizophrenia patients and was common to both the discovery and validation datasets. This consistency across both datasets highlights the potential importance of these genes in the pathophysiology of schizophrenia.

## Functional pathway and molecular function analysis of significant genes in schizophrenia:

**Figure 2:**
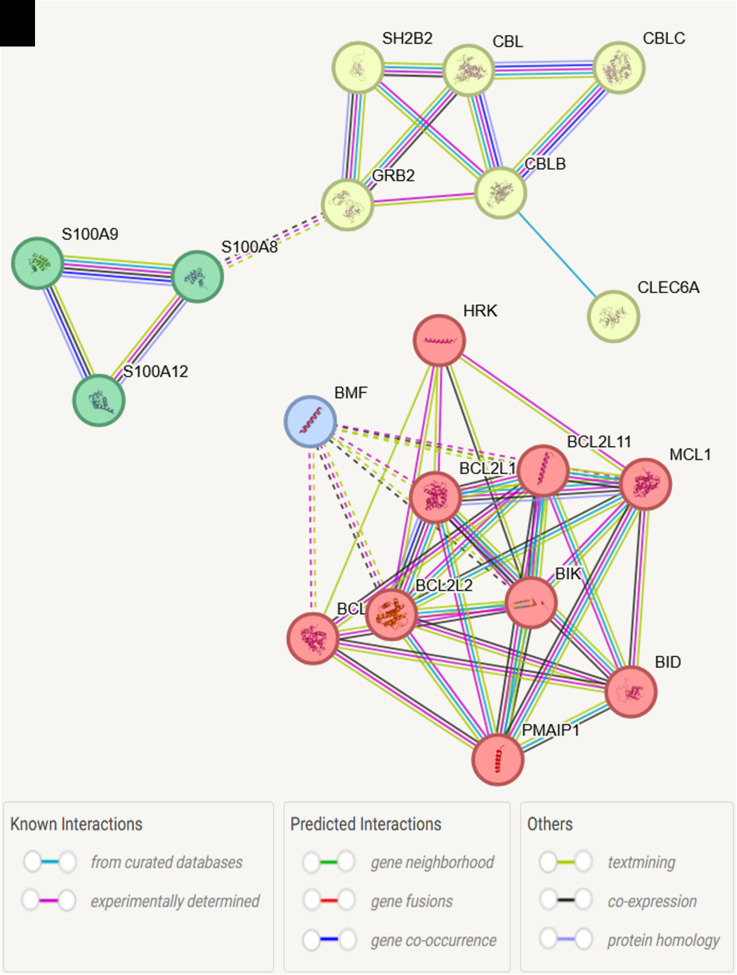
String pathway analysis of the significant genes with k means clustering → Red: apoptosis network (Bcl-2 family protein complex), yellow: Aspartyl transferase activity, Green: S100 protein pathway (1. RAGE receptor binding & 2. Metal sequestration by antimicrobial proteins and Regulation of endothelin production)

**Figure 3:**
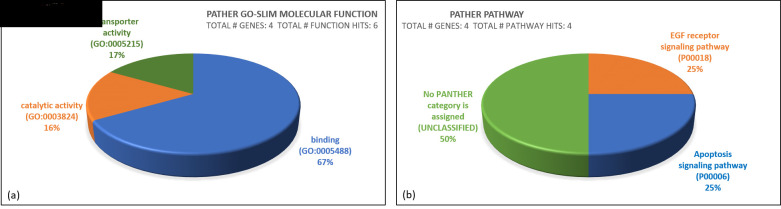
(a) Molecular functions of the significantly altered genes were classified into three categories: binding (n = 4, 66.7%), transporter activity (n = 1, 16.7%) and catalytic activity (n = 1, 16.7%). (n = number of genes, p = percentage). (b) Molecular pathways identified include the EGF receptor signalling pathway and the apoptosis signalling pathway. Source: PANTHER Classification System.

STRING pathway analysis of the significant genes, followed by k-means clustering, revealed distinct interaction networks (Figure 2). The red cluster was associated with the apoptosis network, specifically involving the Bcl-2 family protein complex, emphasizing its role in programmed cell death. The yellow cluster highlighted genes related to aspartyl transferase activity, indicating their involvement in protein modification processes. The green cluster represented the S100 protein pathway, identifying two key functional activities: RAGE receptor binding and metal sequestration by antimicrobial proteins. This cluster was also linked to the regulation of endothelin production, which plays a role in vascular homeostasis and inflammation. These clusters illustrate the functional diversity of the significantly altered genes and their involvement in key pathways relevant to schizophrenia pathology. Additionally, the molecular functions of the significantly altered genes were classified into three main categories: binding (n = 4, 66.7%), transporter activity (n = 1, 16.7%) and catalytic activity (n = 1, 16.7%) (Figure 3) These classifications further emphasize the functional variety within the gene set. Moreover, two key molecular pathways, the EGF receptor signalling pathway and the apoptosis signalling pathway, were identified, providing insights into the biological processes potentially disrupted in schizophrenia.

## Discussion:

The highlight of this study was the identification of four key genes associated with schizophrenia: significant upregulation of S100A8, S100A9 and BCL2A1 and downregulation of CBLB. The upregulation of S100A8 and S100A9 aligns with the role of calprotectin, a heterodimeric protein complex involved in neuroinflammation, in schizophrenia. Additionally, BCL2A1 upregulation is implicated in apoptosis regulation, contributing to grey matter loss in the prefrontal cortex. Conversely, CBLB downregulation may disrupt proteasome-mediated protein degradation, immune regulation and the EGF pathway, potentially leading to cognitive deficits and synaptic dysfunction.

## Upregulation of S100A8 and S100A9 in schizophrenia:

Our study confirms the upregulation of S100A8 and S100A9 in schizophrenia, which has been well-documented in previous research [17, 18-19]. These proteins form calprotectin [17], which plays a critical role in neuro-inflammation, particularly in the prefrontal cortex [18] and hippocampus [20], regions heavily implicated in schizophrenia. Elevated calprotectin levels in these regions may lead to neuronal dysfunction and basilar dendritic loss, contributing to the cognitive impairments seen in schizophrenia patients [21]. The proinflammatory properties of calprotectin, its involvement in pain perception [22] and its dysregulation in peripheral blood mononuclear cells [23] indicate that its role extends beyond the brain, potentially contributing to systemic inflammation. Furthermore, treatment with olanzapine, an antipsychotic drug, has been associated with increased S100A8 and S100A9 expression in the frontal cortex [24], complicating the interpretation of these proteins' role in the disease. This raises the possibility that S100 proteins may influence both schizophrenia pathology and the response to treatment, necessitating further exploration of their multifaceted roles.

## Role of BCL2A1 and apoptosis in schizophrenia:

The upregulation of BCL2A1 in our study underscores the importance of apoptosis regulation in schizophrenia. Bcl-2 family proteins, including BCL2A1, are known to regulate the balance between cell survival and programmed cell death [25]. In schizophrenia, BCL2A1 upregulation in the prefrontal cortex is linked to grey matter loss, which has been associated with cognitive deficits [26]. This finding is consistent with studies showing that schizophrenia patient's exhibit reduced grey matter volume, particularly in the prefrontal regions.

Interestingly, BCL2A1's dual role in either promoting or inhibiting apoptosis depending on the cellular context suggests a complex regulatory mechanism in schizophrenia. Antipsychotic treatments have been shown to elevate Bcl-2 levels, contributing to better outcomes, especially in long-term maintenance therapy [27]. This highlights the protective role of Bcl-2 proteins against cellular damage, potentially offering insights into therapeutic strategies targeting neuroprotection in schizophrenia.

## Downregulation of CBLB and the EGF pathway in schizophrenia:

The downregulation of CBLB, which encodes an E3 ubiquitin-protein ligase, suggests disruptions in proteasome-mediated protein degradation and immune regulation in schizophrenia. The involvement of CBLB in the EGF (Epidermal Growth Factor) pathway points to its potential role in dopaminergic and GABAergic neuron development, which are critical in schizophrenia's neuropathology [28]. Decreased EGF serum levels have been reported in schizophrenia patients [29] and our findings reinforce the role of EGF signalling in the disease. Moreover, Cbl-b is known to regulate synaptic plasticity and memory formation, particularly in the hippocampal regions (CA1, CA3 and the dentate gyrus) [30]. Cbl-b null mice exhibit enhanced long-term memory and short-term plasticity, suggesting that Cbl-b negatively regulates memory. This could be linked to the cognitive deficits observed in schizophrenia patients, where downregulation of CBLB might impair memory and synaptic function. The EGF/ErbB signalling pathway has been extensively studied in schizophrenia, with EGF levels reduced in both the blood and forebrain regions of patients [28, 29]. Abnormal EGF/ErbB signalling persists throughout life and may contribute to several schizophrenia symptoms, particularly those related to cognitive decline and negative symptoms. Targeting this pathway, along with immune modulation, offers a potential therapeutic approach for schizophrenia, as antipsychotics like clozapine have been shown to modulate ErbB receptor kinase activity [31].

## Conclusion:

This study identified key molecular changes in schizophrenia, notably the upregulation of S100A8, S100A9 and BCL2A1 and the downregulation of CBLB, highlighting the involvement of neuro-inflammatory pathways, apoptosis regulation and immune modulation in the disease. The roles of these genes in the prefrontal cortex, critical for cognitive function, emphasize the molecular complexity of schizophrenia and its impact on brain regions central to cognition. Additionally, the potential of S100A8, S100A9 and EGF as biomarkers for disease severity and treatment response opens new possibilities for personalized therapeutic approaches in managing schizophrenia.
